# Examining the educational value of a CanMEDS roles framework in pediatric morbidity and mortality rounds

**DOI:** 10.1186/s12909-014-0262-5

**Published:** 2014-12-16

**Authors:** Donna L Johnston, Anne Rowan-Legg, Stanley J Hamstra

**Affiliations:** Division of Hematology/Oncology, Department of Pediatrics, Children’s Hospital of Eastern Ontario, 401 Smyth Road, Ottawa, ON K1H 8L1 Canada; Division of Pediatric Medicine, Department of Pediatrics, Children’s Hospital of Eastern Ontario, 401 Smyth Road, Ottawa, ON K1H 8L1 Canada; Academy for Innovation in Medical Education, Faculty of Medicine, University of Ottawa, 451 Smyth Road, Ottawa, ON K1H 8M5 Canada

**Keywords:** Morbidity and mortality, CanMEDS, Quality of care

## Abstract

**Background:**

In order to determine whether the CanMEDS roles could be helpful in solidifying knowledge during clinical training, we examined quality of care issues identified during morbidity and mortality (M&M) rounds.

**Methods:**

During the M&M rounds, following the case presentation, there was a pause and attendees were asked to identify quality of care issues that were present in the case. The attendees were assigned to a CanMEDS prompted group or non-prompted group. Following the rounds, the issues were identified, coded according to CanMEDS role, and compared between groups.

**Results:**

A total of 111 individuals identified a total of 350 issues; 57 individuals were in the CanMEDS-prompted group and 54 were in the unprompted group. The mean number of issues identified was significantly higher in the CanMEDS-prompted group compared to the unprompted group (3.7 versus 2.6, p = 0.039). There were significantly more issues raised in the prompted group for the roles of communicator, collaborator, scholar and professional.

**Conclusions:**

Using CanMEDS roles as prompts, attendees at M&M rounds identify more quality of care issues than if not given a prompt. Use of the CanMEDS framework may assist learners to consolidate the linkage between expected training objectives and the complexities of clinical practice.

## Background

Morbidity and Mortality (M & M) rounds have the potential to serve as an excellent learning experience for all heath care providers and can be used to teach about health care system process, patient safety issues, and quality of care and improvement [1]. M & M rounds are used routinely in many academic health centers, including pediatrics, but there is limited literature examining the role of M & M rounds in enhancing the educational experience of its participants. Studies demonstrate that the M & M conference can be used as a forum for teaching the Accreditation Council for Graduate Medical Education (ACGME) general competencies [1,2], general surgery curricula [3], and family medicine curricula [4]. The majority of the literature on M & M rounds pertains to surgical M & M rounds [1-5]. A 2005 study described the M & M review process at Canadian pediatric centers and found that the process and standards for case review were inconsistent [6]. This study made recommendations for standards and stricter definitions of morbidity and mortality. Little has been published on the progress of pediatric M & M rounds since that time.

Like the ACGME competencies, the CanMEDS framework is a roles framework that was designed to assist residency programs in training all aspects of clinical practice, with the goal of improving patient care [7]. The CanMEDS framework articulates a comprehensive definition of the competencies needed for medical education and practice, organized within seven defined roles: manager, collaborator, communicator, scholar, medical expert, health advocate and professional. These seven roles form the basis of medical education curriculum; residents are expected to be competent in all roles by completion of training and are assessed based on these competencies.

The objective of this study was to determine whether the provision of prompts of the CanMEDS roles improves the number and type of quality of care issues identified by the M & M rounds participants.

The M & M rounds traditionally focus on analyzing the medical expert aspects of the selected case [1]. This role is identified as the central role for the CanMEDS competencies [7]. Thus we expected that in identifying quality of care issues during M & M rounds, this role would have the most identified quality of care issues and would not be dependent on prompting of the CanMEDS roles.

It was our belief that M & M rounds have the potential to serve as excellent educational tools for examining other, non-medical expert competencies. In using the CanMEDS framework to identify other issues of patient care, prompted attendees may expand consideration to other non-medical expert roles enhancing a critical and thorough examination of a case and how they impact patient care.

## Methods

The Children’s Hospital of Eastern Ontario (CHEO) holds monthly M & M Rounds in the Department of Pediatrics. These rounds are traditionally attended by medical students, residents, fellows (subspecialty residents) and departmental staff pediatricians. A senior pediatric resident presents the case to be reviewed. Currently there is no prescribed structure for the M & M rounds, though at CHEO the case conference traditionally includes a presentation of the case followed by a discussion raising the quality of care issues specific to the case. Recommendations for improvement are subsequently generated.

**Figure 1:**
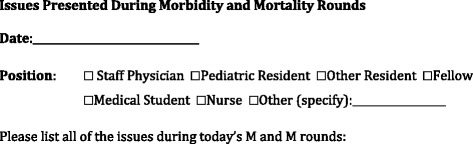
**Data collection sheet for unprompted group.**

**Figure 2:**
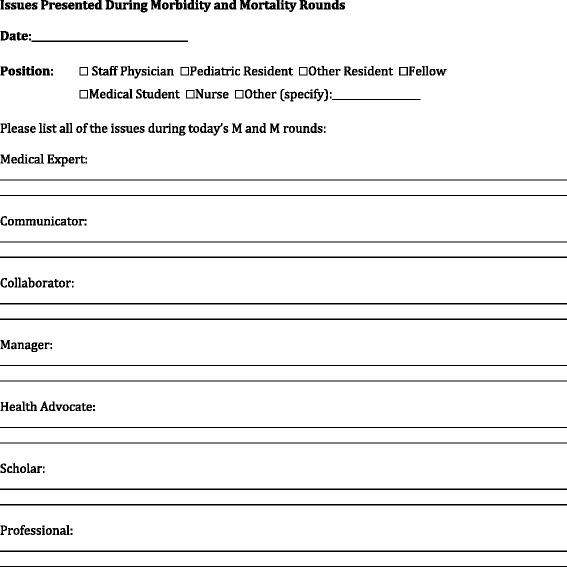
**Data collection sheet for CanMEDS prompted group.**

In this study, at the beginning of rounds all attendees were told that a study on M & M rounds was being done and were asked to participate. Following the case presentation (and before the discussion), all attendees of the audience were asked to identify quality of care issues that they felt were important and relevant to the case. (The M & M Rounds currently have no formal process, nor are attendees given specific training with which to identify quality of care issues). Each attendee was given a paper on which to write quality of care issues they could identify. The attendees were randomized to one of two groups based on which side of the aisle they sat on. Half the attendees received a sheet asking to list all the quality of care issues they felt pertinent to the case (Figure 1). The other half of the attendees received a sheet asking to list the quality of care issues pertinent to the case with the headings of the 7 CanMEDS roles (Figure 2). These forms were completed by the attendees (with a period of about 10 minutes allotted) and then collected prior to engaging in the discussion of the case. The side of the room the different forms were placed on was alternated between rounds.

Following the rounds, the investigator counted the issues that each attendee identified and entered the data into a database. The CanMEDS role chosen by the attendee for their quality of care issue was not modified. Thus some issues, such as scholar and medical expert could have similar issues depending on where the attendee felt it was most applicable. For the unprompted group, the issues were first coded by CanMEDS role based on the role best fitting the issue, by the investigator prior to entering into the database.

This process was repeated at four M & M rounds, each 2 months apart. The residents and medical students rotate every four to eight weeks, so an interval of 2 months ensured some new attendees at the rounds. As well, this was felt to prevent contamination if an attendee was placed in a different group during subsequent data collection times. Also, the attending staff involved in the case usually attended the rounds, so by having different types of cases being presented, new attending physicians attended the rounds.

This study was approved by the CHEO Research Ethics Board. There was implied consent from participants by completing the form.

The data were entered into a database, and ANOVA performed using SPSS software (version 19). A p-value less than 0.05 was considered statistically significant.

## Results

Data were collected at M & M rounds in July, September, November 2011, and January 2012. The first M & M rounds involved a patient with a complicated congenital cardiac abnormality with a delayed diagnosis. The second M & M rounds involved a child with immune deficiency with investigations that were lost to follow up resulting in a delayed diagnosis. The third M & M rounds involved a child with an anion gap acidosis found to be secondary to methanol ingestion. The fourth M & M rounds involved a delay in diagnosis of a thalamic tumor causing diabetes insipidus. Examples of identified quality of care issues for each case are described in Table 1. There were no responses that were not meaningful to the case or duplicative responses on the same response sheet.

**Table 1:** **Examples of quality of care issues identified at each M & M rounds**

**CanMEDS role**	**Rounds topic**			
	**Cardiac abnormality**	**Immune deficiency**	**Acidosis**	**Thalamic tumor**
Medical expert	Missed cardiac anomaly	Not recognize symptoms of immunodeficiency	Lack of consideration of toxic ingestion as possible cause	Interpretation of laboratory results
				Diabetes insipidus management
Communicator	Explanation to parents re severity/bad news	Coordination between subspecialties	Transfer of information between services	Poor interprofessional communication
			No phone call from lab with abnormal methanol level	Poor disclosure of information to parents
Collaborator	Collaboration between multiple disciplines	Referral to general pediatrics when outside scope of subspecialty	Collaborating with other teams re differential diagnosis and labs	Collaboration with radiology to have MRI done urgently
Professional				Good notekeeping needs to be done especially about information/conversations with family
Health advocate		Ensuring comprehensive continuity of care	Advocacy for investigating other diagnoses	Pushing for earlier investigations if deemed appropriate
Scholar		Considering differentials for lymphadenopathy/splenomegaly	Knowledge of methanol ingestion signs	
Manager	Time to operation	Who is in charge of results		Asking for imaging earlier if high likelihood of mass
		Lack of follow up of abnormal results		

The majority of individuals who participated were residents (64) followed by staff physicians and medical students (20 each). There were no nurses who participated in the study and one laboratory technician participated (listed as other in table). A total of 111 individuals participated and identified a total of 350 quality of care issues in the study; 57 individuals were in the CanMEDS-prompted group and 54 individuals were in the unprompted group. The number of issues raised per rounds was: round 1: 23, round 2: 22, round 3: 31 and round 4: 35. The mean total number of issues identified was significantly higher in the CanMEDS prompted group compared to the unprompted group (3.7 issues versus 2.6 issues, p = 0.039). The number of issues raised based on each role are shown in Table 2. There were significantly more issues raised in the prompted group for the roles of communicator, collaborator, scholar and professional, and no significant differences in the roles of medical expert, manager and health advocate.

**Table 2:** **Number of quality of care issues raised per CanMEDS role**

**CanMEDS role**	**Prompted group**	**Unprompted group**	**P Value**
Medical expert	48	40	0.167
Communicator	49	31	0.009
Collaborator	26	1	0.001
Manager	21	14	0.813
Health advocate	20	11	0.122
Scholar	7	0	0.007
Professional	5	0	0.026
Total number of quality of care issues	176	97	0.039

When examining the quality of care issues raised in each CanMEDS role, the mean number of issues raised per role varied from 2.0 to 3.5 (Table 3). There was no difference in the number of quality of care issues raised in each CanMEDS role based on hospital position (medical student, resident, staff, etc) (p = 0.129-0.908).

**Table 3:** **Number of quality of care issues for different hospital position**

**Hospital position**	**Number of individuals**	**Number of issues identified**	**Mean number of issues identified per individual**
Staff	20	68	3.4
Pediatric resident	55	191	3.5
Other resident	9	21	2.3
Fellow	6	13	2.2
Medical student	20	55	2.8
Nurse	0	0	0
Other	1	2	2
Total	111	350	3.15

## Discussion

There were significantly more quality of care issues identified when attendees were prompted with the CanMEDS roles than when unprompted. As expected, the medical expert role had the most number of issues identified when combining both the prompted and unprompted groups [1], and there was no significant difference in the number of issues related to the medical expert role between the two groups.

With respect to the other CanMEDS roles, there was a significant difference in the number of issues generated by the prompted and unprompted groups in the roles of communicator, collaborator, scholar and professional, but not with manager and health advocate. The fact that manager and heath advocate roles were not significantly different may be secondary to the nature of the cases that were presented, having multiple quality of care issues involving these roles central to the case. It may also be due to the fact that these roles are emphasized during residency training.

The fact that four roles were significantly different between the two groups of attendees suggests that quality of care issues involving these roles were less obvious to attendees, or alternatively may not have been central to the case. The former may suggest a need for more emphasis during training.

There was no difference in number of quality of care issues raised based on hospital position which suggests that length of time from training (medical student versus resident versus subspecialty fellow versus staff physician) does not affect identification of these issues. Thus the potential benefit of training emphasis of these CanMEDS roles spans from medical school to continuing medical education for practicing physicians.

The limitations of this study include the fact that the prompted group may have generated quality of care issues based on simply having prompts, not necessarily because they were CanMEDS roles. This limitation would have been overcome by having a third group with a different set of prompts, however what prompts to include that were not included in the 7 CanMEDS roles is a challenge. The CanMEDS roles describe the knowledge, skills and abilities that specialist physicians need for better patient outcomes and are based on the seven roles that all physicians need to have to be better doctors [7]. Thus this study only assesses comparison with the CanMEDS competencies as prompts as we felt they encompass the majority of prompts to cover all aspects of potential issues related to the case. As well, there may have been an effect of time constraints for a participant to record the issues related to the case. Further, the number of issues generated does not necessarily speak to their quality, or whether the number of issues generated leads to better discussion and subsequent recommendations from M & M rounds. Another limitation would include the participant’s knowledge and familiarity with the meaning of each of the CanMEDS roles. As well, each M & M case may not have raised particular issues or concerns for each of the individual CanMEDS roles. Lastly, there was not true randomization into each group, but assignment was based on which side of the room the attendee sat on, thus restricting the generalizability of the conclusions somewhat. A future study should be conducted with true random assignment between groups to rule out any underlying selection bias.

A recent study of pediatric M & M rounds examined restructuring the rounds and resulted in enhanced education, satisfaction and quality improvements [8]. Another recent study of pediatric M & M rounds standardized the case review and found that this enhanced patient safety and quality improvement [9]. Based on this recent literature and on our observations, presenting M & M rounds using a framework of the CanMEDS roles could be a valuable format for issue generation. It has been noted that there is a need for standards and stricter definitions of morbidity and mortality rounds [6]. For future study, we plan to develop a format for the rounds based on CanMEDS roles and then investigate if there is a change in the quality of discussion and recommendations generated following a change in the M & M rounds process.

## Conclusions

Overall this study demonstrated that using the CanMEDS roles as prompts, attendees at M & M rounds identify more quality of care issues than if not given a prompt. Use of the CanMEDS framework may assist learners to consolidate the linkage between expected training objectives and the complexities of clinical practice. This has the potential to enhance the educational effectiveness of M & M rounds.
